# When experience does not promote expertise: security professionals fail to detect low prevalence fake IDs

**DOI:** 10.1186/s41235-021-00288-z

**Published:** 2021-04-01

**Authors:** Dawn R. Weatherford, Devin Roberson, William Blake Erickson

**Affiliations:** Psychology Program, Department of Life Sciences, Texas A&M University-San Antonio, 1 University Way, San Antonio, TX 78224 USA

**Keywords:** Low prevalence effect, Facial comparison, Facial identification, Professional experience, Facial reviewers

## Abstract

Professional screeners frequently verify photograph IDs in such industries as professional security, bar tending, and sales of age-restricted materials. Moreover, security screening is a vital tool for law enforcement in the search for missing or wanted persons. Nevertheless, previous research demonstrates that novice participants fail to spot fake IDs when they are rare (i.e., the low prevalence effect; LPE). To address whether this phenomenon also occurs with professional screeners, we conducted three experiments. Experiment 1 compared security professional and non-professionals. Experiment 2 compared bar-security professionals, access-security professionals, and non-professionals. Finally, Experiment 3 added a newly created Professional Identity Training Questionnaire to determine whether and how aspects of professionals’ employment predict ID-matching accuracy. Across all three experiments, all participants were susceptible to the LPE regardless of professional status. Neither length/type of professional experience nor length/type of training experience affected ID verification performance. We discuss task performance and survey responses with aims to acknowledge and address this potential problem in real-world screening scenarios.

**Supplementary Information:**

The online version contains supplementary material available at 10.1186/s41235-021-00288-z.

## Background

In response to various worldwide security concerns at borders and other ports of entry, professional screeners commonly restrict access of goods and services to authorized individuals who present an authentic ID card. However, a potential traveler may produce stolen, borrowed, or inauthentic documents. Professional screeners need to maintain safety by identifying such imposters, while still allowing lawful passengers through. Although technological advancements such as automatic face recognition systems (e.g., Taigman et al., 2014) and various methods of biometric scanning may seem like attractive alternatives to replace human screeners, such technologies face many of the same challenges as human recognizers (O’Toole et al., 2012; e.g., Tran et al., 2017), while also raising concerns about ethics, transparency, and accountability (Drozdowski et al., 2020). Therefore, the bulk of imposter detection duties has been and is being performed by humans.

Even under optimal viewing conditions, ID matching performance has a surprisingly high number of errors (e.g., Burton, 2013). Errors further increase with additional real-world challenges such as time pressure (e.g., Bindemann et al., 2016) and vigilance (e.g., Alenezi et al., 2015). Among a host of challenges to successful ID screening, the *Low Prevalence Effect* (LPE; e.g., Wolfe et al., 2007) also increases error rates. As a well-known cognitive phenomenon, the LPE has been demonstrated for infrequent targets such as objects in a visual array (e.g., Fleck & Mitroff, 2007), weapons in luggage X-rays (Wolfe et al., 2013), and most relevant to the current investigation, fake IDs (Papesh et al., 2018; Papesh & Goldinger, 2014; Susa et al., 2019; Weatherford & Schein, 2015; Weatherford et al., 2020; cf. Bindemann et al., 2016). For instance, Weatherford et al. (2020) tested untrained participants’ ability to make identity judgments about a target face presented beside an ID card. Fake IDs appeared in either 10%, 50%, or 90% of all trials. Consistent with the LPE, participants inaccurately accepted more fake IDs (i.e., mismatch errors) in the 10% prevalence condition. What’s more, performance feedback only exacerbated the effect.

Of theoretical interest, research suggests that the LPE is caused by early search termination (i.e., making a decision before exhausting all available cues) and/or criterion shifting (i.e., response criterion, as defined by signal detection theory, shifts over the course of trials). Although work outside of facial identification has demonstrated that early search termination might be corrected by allowing participants time to reconsider rash decisions (e.g., Fleck & Mitroff, 2007), ID matching tasks have consistently failed to find any evidence for early search termination (Papesh & Goldinger, 2014; Weatherford et al., 2020). In contrast, facial studies more strongly favor criterion shifting that biases acceptance of fake IDs as authentic even with additional consideration time, warnings about errors, bursts of high-prevalence mismatch trials, and other manipulations designed to combat criterion shifting. In other words, the LPE is an exceptionally stubborn source of errors for which we need a greater theoretical understanding.

Although LPE tasks in the laboratory (e.g., Papesh & Goldinger, 2014; Weatherford et al., 2020) may be cautiously generalized to other real-world ID matching tasks, no studies have directly tested if and whether the LPE emerges in an ID matching task with a professional sample. It is quite possible that this phenomenon is an artifact of the untrained participants performing tasks with which they may have little to no experience. Although it is difficult to estimate exactly how often professionals are presented with fake IDs, security screeners are likely far more accustomed to seeing an authentic ID card presented by its rightful owner, and that expectation may influence performance on an ID matching task.

Therefore, the current studies report data from professional samples on an ecologically valid variant of a routine task for which they have extensive experience. We hope that results will illuminate if professionals may commit LPE errors in a facial identification task.

## Mapping a laboratory-based task onto a professional-security setting

To increase ecological validity and reduce the influence of potential confounds associated with laboratory-based designs, we modeled our ID matching task to reflect essential aspects of a professional-security setting.

### Realistic document images

During an ID check in the real world, a screener is responsible for satisfying two goals. One goal involves authenticating the individual’s documents by visually scanning for particular security features such as expiration dates, ghost images, and black-light responsive materials. Many different agencies focus exclusively on this aspect of the screening process. Continually updated patents reflect that improvements in this area focus on combating the passage of fraudulent documents as a measure to heighten security and stop criminal behavior. The steps needed to complete this goal are relatively straightforward, and screeners are sometimes provided with tools (e.g., black-light scanners) to aid detection of fake IDs. Therefore, we reduced participants’ attention to this goal by presenting standardized document images for which security features (e.g., expiration dates, ghost images) were either removed or held constant to reduce possible suspicion of fraudulent documents.

A second goal involves authenticating an individuals’ identity by comparing the facial image to the person presenting it. The steps needed to complete this goal are far more nebulous—but equally, if not more, important. A successful screener needs to not only catch fraudulent documents (e.g., presented after printed expiration date), but also fraudulent identities (i.e., presented by an imposter). As the more abstract of the two tasks, this second goal of facial image comparison has been the subject of extensive cognitive investigations to reveal the underlying mechanisms that predict (or fail to predict) success.

### Realistic facial images

Research suggests that although humans possess superior facial recognition skills with familiar faces (e.g., Kramer et al., 2018; Young & Burton, 2018), this expertise does not completely extend to unfamiliar faces (Abudarham et al., 2019; Burton, 2013; Dunn et al., 2018) that would be typical for a security-screening scenario. Although both processes share some perceptual characteristics (e.g., featural analysis, perceptual sensitivity; Abudarham et al., 2019), many of the hallmarks of familiar face processing revolve around more well-developed conceptual/associationistic processing that increases the screener’s ability to generalize from one image of someone to many others. In other words, facial image comparison only plays a crucial role when screeners need to authenticate the documents of unknown individuals.

Many agencies that produce IDs attempt to reduce or eliminate perceptual variations that impair unfamiliar face comparison (e.g., Hancock et al., 2000). These variations including suboptimal lighting/shadows (e.g., Braje et al., 1998), distance (e.g., Lampinen et al., 2014), pose (e.g., Hancock et al., 2000), image quality (e.g., Bruce et al., 2001), and partial face coverage (e.g., Davies & Flin, 1984). Accordingly, all facial images used in this series of experiments depict a frontal pose of each participant under adequate lighting without any obstructions (e.g., sunglasses, facial scarves). Further, images were of similar size and quality to those used in typical screening scenarios.

### Realistic target comparison images

Even when presented with a facial images are optimized, the screening task remains challenging because a person may look markedly different from their own ID photograph. Variability over time (e.g., caused by a change in hair, weight, or cosmetic alterations) increases *within-person* variability. Recently, Weatherford et al. (2020) investigated the relationship between within-person variability and the LPE. Over the course of three experiments, the authors replicated the classic LPE, such that participants in the low mismatch prevalence condition demonstrated lower mismatch accuracy than those in medium and high mismatch prevalence conditions. As the *within-person* variability in each experiment was increased through the use of photographs that were captured further apart in time (i.e., from same day images in Experiment 1 to images taken at least one year apart in Experiment 3), the LPE only became more pronounced. Therefore, we removed the possibility that the task would be trivially simple by using target comparison images/videos taken in a different context, with a different camera, and that varied in time by at least six months from the facial image on the document.

## How professional screening experiences affect the LPE

After establishing ecologically valid measures, the current study extends beyond student samples reported in previous studies (Papesh et al., 2018; e.g., Papesh & Goldinger, 2014; Susa et al., 2019; Weatherford et al., 2020) by recruiting ID screening professionals. Aside from training, which has shown mixed results in improving ID screening accuracy (e.g., Towler et al., 2014, 2019), professionals’ routine experience with ID screening may affect ID matching accuracy. As no published work has examined the relationship between professional experience and the LPE, two theoretically supported, yet divergent, possibilities may predict facial image comparison performance.

### Professional experience might reduce LPE errors

In a practical sense, the best-case scenario would involve professional improvements in facial comparison performance alongside reductions in mismatch LPE errors. If experience promotes expertise, then a wide variety of security professionals should outperform naive, untrained participants on both the task itself (i.e., overall accuracy), and the detection of fake IDs (i.e., mismatch error rate). In the present studies, we focused on facial reviewers (i.e., individuals who perform a high volume of routine facial comparison tasks within a relatively short period of time throughout their entire shift; see also FISWG, 2011), as these professionals perform the bulk of identification matching operations designed to ensure public safety. Occupations such as police officers, security guards, border patrol agents, and the like would fit within this category.

Concerning overall accuracy, some studies have demonstrated that professional experience improves matching performance (i.e., overall performance on match and mismatch trials). These studies typically appeal to benefits by way of quantitative (e.g., years of employment) and qualitative (e.g., type and amount of identity comparisons made across a variety of contexts) aspects of professional experience. One illustrative example of improved performance involved the comparison of passport issuance officers to novices in a photograph-to- photograph-ID matching task. Using a relatively large sample size (*n* = 204), Towler et al. (2019; Experiment 2) found that professionals outperformed novices on a photograph-to-photograph task using unfamiliar faces, with benefits modestly increasing in line with the difficulty of the tasks. Similar findings using other photograph-to-photograph comparisons have also been observed (e.g., Phillips et al., 2018).

### Professional experience might not affect, or might exacerbate, LPE errors

While the best-case scenario is attractive, the larger body of evidence supports the possibility of a non-significant finding. More specifically, several studies assessing facial reviewers’ skills (e.g., White et al., 2014; Papesh et al., 2018Heyer et al., 2018; Wirth & Carbon, 2017) were not as successful. Professional experience did not improve task performance. If the current studies fit within this body of work, then we might predict no benefit of professional experience.

One last theoretical possibility is that professional experience will *exacerbate* LPE errors. In the field, it is reasonable to expect that professionals see a low prevalence of fake IDs. This low prevalence likely shifts their criterion, thereby tempting a screener to misattribute a high variability between and ID and its presenter as *within-person* variability (i.e., the differences seen between two images of the same person) instead of *between-person* variability (i.e., the similarities seen between two images of different people). In other words, when individuals seeking access to restricted goods and services present an ID with large differences (e.g., glasses, aging effects, cosmetics), the most practical approach would be to assume that variability is a natural by-product of comparing an individual to an image from up to ten years ago. If fraudulent documents and identities are rare, this heuristic would serve to reduce false alarms (i.e., rejecting an authentic ID). To the extent that professional experience transfers to other identity matching tasks, and those heuristics and assumptions remain intact, then professionals may actually commit more LPE errors than untrained novices. Given that several studies have found criterion shifting even within a short experimental period (e.g., Papesh et al., 2018; Susa et al., 2019; Weatherford et al., 2020), it is entirely reasonable that routine, professional experiences could negatively transfer to this new task.

## The current studies

In three studies, we manipulated the ratio of fake to authentic IDs across participants and expected to replicate previous findings with our non-professional participants. In the low mismatch condition (i.e., 90% matches to 10% mismatches), we predicted that non-professionals would identify fake IDs less often. Likewise in the low match condition (i.e., 10% matches to 90% mismatches), we predicted that non-professionals would identify authentic IDs less often. We expected that these differences, compared to a balanced condition (50% matches to 50% mismatches), would be largely driven by criterion shifting. In other words, participants in the imbalanced conditions would either relax their criteria for a “match” decision under the low mismatch prevalence or constrain their criteria for a “match” decision under low match conditions.

To foreshadow our findings, all of our predictions were confirmed for non-professionals. Furthermore, despite possible differences to task performance that might be expected with professional security experience, professional status did not affect our pattern of results. Participants in both types of imbalanced groups (low mismatch and low match) produced the typical pattern of the LPE. Even though feedback may have heightened awareness of the imbalanced base rates (a point we revisit in the General Discussion), the data consistently support no differences in ID verification performance across professional status, type of security occupation, and self-reports of previous employment and training histories.

## Experiment 1

### Method

#### Participants

##### Recruitment

All participants were recruited through a university subject pool or a Qualtrics panel (www.Qualtrics.com). Qualtrics recruiting ensured that participants’ self-reported age, gender, race/ethnicity, and income generally reflected established patterns reported in the 2010 US Census. For specific qualifications, such as professional security experience, Qualtrics panels use targeted marketing to attract participants from specific partners with whom they establish relationships and subsequently confirm the availability of participants with the requisite qualifications for inclusion. Understandably, many of the professionals might have been hesitant to provide the name of their employer. And, to protect their identities under such circumstances as to encourage their honest responding, we did not solicit that specific information.

##### Sample

The sample consisted of *N* = 78 individuals. Non-professionals (*N* = 54; *M*age = 37.22 years, SD = 14.53; 4 failed to report; 38 females) participated in exchange for partial course credit or $6.00 (all compensation rates reported in USD),^1^ depending upon recruiting source. Professionals (*N* = 24; *M*age = 48.3 years, SD = 13.79; 4 failed to report; 7 females) reported at least 1 year of professional ID card screening experience and participated in exchange for between $20 and $30. Both groups represented a diverse sample of participants (59.0% White/Caucasian, 26.9% Hispanic/Latinx, 11.5% Black/African-American, 0% Asian/Pacific Islander, and 2.6% other).^2^

#### Power analysis

In order to determine the appropriate sample size for our design, we referred to the documented effect sizes in Weatherford et al. (2020). To capture the interactive effect of mismatch prevalence and feedback, we used effect sizes from Experiment 2, which used the same stimuli as we adopted here: *η*^2^ *p* = 0.16 (with subsequent interpretation of values above 0.25 being large, 0.09 being medium, and 0.01 being small) which converts to *f* = 0.44 (with 0.40 considered large, 0.25 considered medium, and 0.10 considered small; Cohen, 1988). We conducted an a priori power analysis in G*Power (Faul et al., 2007) using the statistical test for “ANOVA: Fixed effects, special, main effects and interactions” using desired power (1 − β probability) of 0.90 and a numerator *df* of 2. G*Power indicated a required sample size of *n* = 70. Thus, our sample size of *n* = 78 exceeded the sample size necessary to achieve sufficient power.

#### Materials

##### Identity matching task

**Fig. 1:**
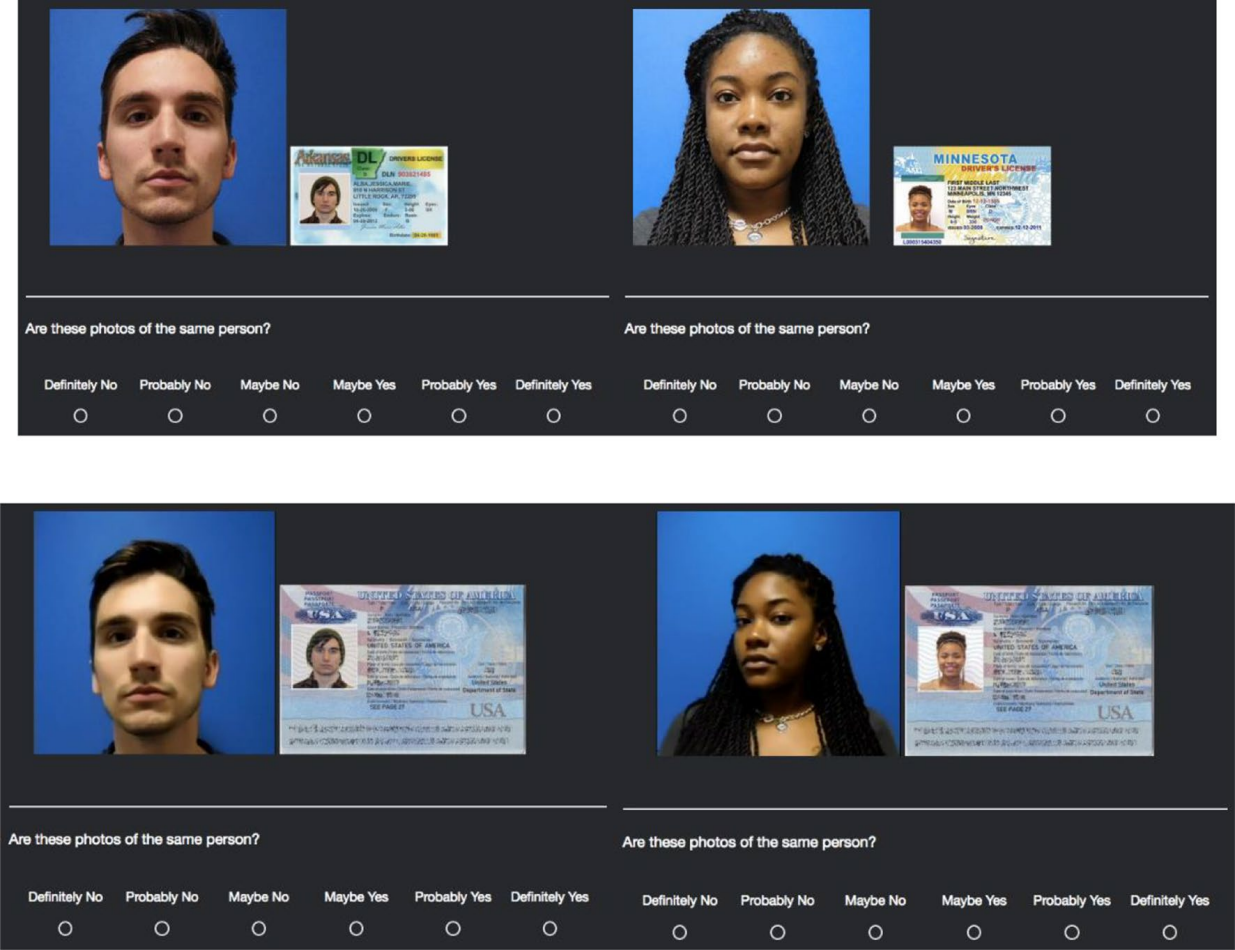
Example stimuli of identities used in experiment 1 (top row) and experiments 2 and 3 (bottom row). Experiment 1 presented a still target image compared to a state-based photograph ID. Experiments 2 and 3 presented a moving target video compared to a US passport. The left column represents match pairs, whereas the right column represents mismatch pairs. All images used with permission

Participants viewed 200 unique images of 100 identities from a facial database (Weatherford et al., 2016) that approximated the same racial/ethnic and gender composition as the 2010 US Census. Each trial displayed a static target image (296 × 296 pixels) beside another static image embedded in one of six state ID templates (200 × 120 pixels) on a black background (see Fig. 1). All static images were taken in a controlled environment. The target image was taken in front of a blue background, and the state ID image was taken in front of a white background. The two images were taken with different cameras, in different settings, between six months to five years apart.

All image pairs were the same as described in Weatherford et al. (2020), Experiment 2. Among 100 identity pairs, we randomly chose 10 to serve as low-prevalence targets for the imbalanced conditions (i.e., presented as mismatches for the low mismatch prevalence condition or matches for the low match prevalence condition). For the balanced prevalence condition, we randomly split the 100 pairs across two different versions (i.e., the 50 identity pairs that served as mismatches in one counterbalance variation were displayed as matches in the other counterbalance variation). All identities were fully counterbalanced across all conditions, such that no participant viewed the same image more than once (for complete counterbalancing legends, see OSF website listed below).

#### Design and procedure

All participants accessed the ID matching task and provided informed consent online through Qualtrics. Participants were randomly assigned to one of three prevalence conditions (low mismatch/10% mismatches; balanced/50% mismatches; or low match/90% mismatches) and three feedback conditions (full feedback, error-only feedback, or no feedback). On each of the 100 trials, participants responded to the question “Are these photographs of the same person?” on a 1–6 scale, with 1 indicating *Definitely No* and 6 indicating *Definitely Yes*. After making the decision, participants in the full feedback condition saw one of four feedback statements. After a correct decision, they viewed “You rejected a fake ID” for mismatch trials or “You accepted an authentic ID” for match trials. After an incorrect decision, they viewed “You accepted a fake ID” for mismatch trials or “You rejected an authentic ID” for match trials. Participants in the error-only feedback condition only received the feedback statements after incorrect decisions. Participants in the no feedback condition did not see any statements about their performance. After 100 trials, participants provided demographic information, were debriefed, and thanked for their time.

## Results

**Fig. 2:**
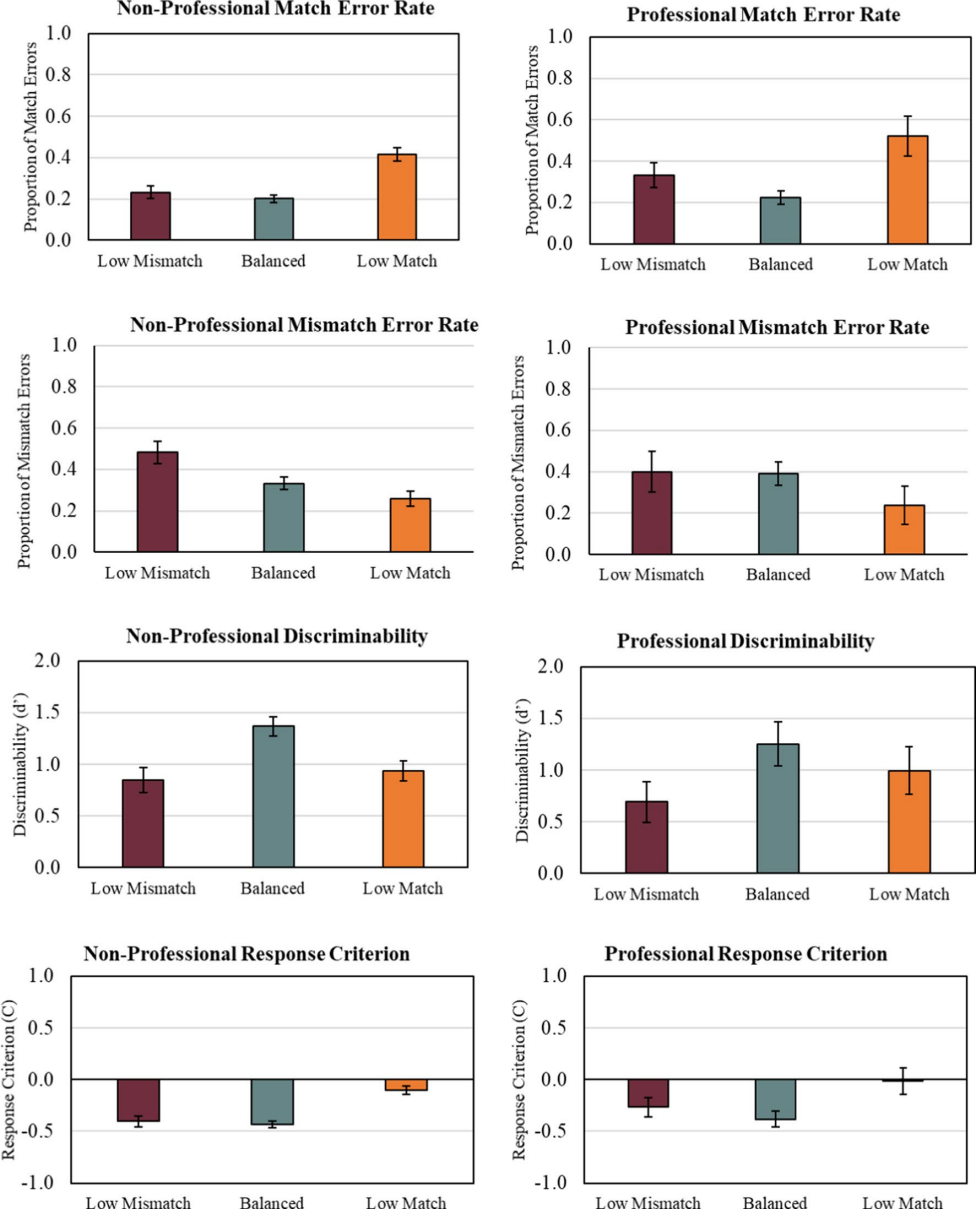
Average non-professional (left column) and professional (right column) match error rate, mismatch error rate, discriminability, and response criterion at each level of prevalence collapsed across feedback conditions from experiment 1. Error bars represent standard error of the mean (SE)

Our first experiment supports the conclusion that professionals exhibit the LPE, following the same patterns as non-professionals, regardless of what type of trial is infrequent. In other words, when fake IDs were infrequent—mismatch errors rose. When authentic IDs were infrequent—match errors rose. Further, the data support a criterion shift explanation, as evidenced in Fig. 2 (see also, Weatherford et al. 2020).

### Analysis of discriminability and response criterion

We first binarized our data into match/mismatch decisions by collapsing across the 1–6 options (see Stanislaw & Todorov for comparisons of rating tasks with yes/no tasks^3^). We coded yes responses (i.e., Definitely Yes, Probably Yes, and Maybe Yes) to the question, “Are these photographs of the same person?” as match decisions. We coded no responses (i.e., Definitely no, Probably No, and Maybe No) as mismatch decisions. Therefore, correct responses to match and mismatch trials were coded as hits and correct rejections, respectively. Incorrect responses to match and mismatch trials were coded as misses and false alarms, respectively (although see Papesh, 2014, for an alternative coding approach that treats successful identification of mismatches as “hits”). To account for extreme performance levels (e.g., hit or false alarm rates of zero), extreme values were replaced by 1–2/*N* for rates of 1 or 2/*N* of 0, where *N* represents the number of trials of that type. For ease of comparison, the complementary error rates for misses and false alarms are depicted in Fig. 2.

We further went on to analyze signal detection measures of discriminability (*d*′) and response criterion (*C*). For *d*′, higher values are interpreted as a greater ability to distinguish between match and mismatch trials. For *C*, a value of zero represents no bias toward either match or mismatch decisions. However, lower or negative *C* values are interpreted as a greater likelihood of making a match decision. In contrast, higher *C* values are interpreted as a greater likelihood of making a mismatch decision.

We conducted a 3 (Prevalence: Low Mismatch, Balanced, Low Match) × 3 (Feedback: None, Error, Full) × 2 (Group: Professional, Non-Professional) factorial analysis of variance on discriminability measure d'. As can be seen in Fig. 2, the ANOVA revealed that only Prevalence significantly affected discriminability, *F*(2, 58) = 5.61, *p* = 0.006, *n*^2^*p* = 0.160. Tukey's Bonferroni-corrected HSD found that Balanced prevalence yielded greater discriminability than Low Match prevalence (*p* = 0.02) and Low Mismatch prevalence (*p* = 0.003), which were not significantly different from one another. No other factors yielded significant main effects or interactions.

We next conducted a 3 (Prevalence: Low Mismatch, Balanced, Low Match) × 3 (Feedback: None, Error, Full) × 2 (Group: Professional, Non-Professional) factorial analysis of variance on response criterion measure C. As predicted, Prevalence significantly affected criterion, *F*(2, 58) = 14.78, *p* < 0.001, *n*^2^*p* = 0.334. Tukey's Bonferroni-corrected HSD found that criterion under Low Match prevalence was significantly higher than Balanced (*p* < 0.001) and Low Mismatch prevalence (*p* < 0.001), which were not significantly different from one another. Criterion for professionals was marginally higher than non-professionals, *F* (1, 58) = 2.91, *p* = 0.09. These effects were accompanied by a significant Prevalence x Feedback interaction, *F*(4, 58) = 4.63, *p* = 0.003, *n*^2^*p* = 0.239. Planned follow-up comparisons found this interaction was driven by significant univariate effects of Feedback at Low Mismatch prevalence [*F*(2, 59) = 5.47, *p* = 0.007, *n*^2^*p* = 0.157], but not other levels of prevalence.

## Discussion

Although these initial results seem promising, we sought to replicate and extend them to a modified paradigm with more externally valid materials. We also differentiated between different types of professional groups, as opposed to simply treating all professional experiences similarly.

Regarding the paradigm modifications, Experiments 2 and 3 were altered by replacing a) the static target image with a video of a person in a frontal pose rotating their head from side to side, and b) the state-based driver license templates with US passports. First, we justified the choice to incorporate video targets by relying upon previous research comparing still to moving images (e.g., Pike et al., 1997; Zhao & Bülthoff, 2017). Videos create a richer encoding experience that allows a screener to consider additional cues that are unavailable in a static image. These additional cues might then become diagnostic evidence to support a more informed match or mismatch decision (e.g., Pilz et al., 2006). At a theoretical level, the extant literature in facial identification and recognition supports both qualitative and quantitative differences that confer advantages in processing for moving versus still images (Lander et al., 1999; O’Toole et al., 2002; Thornton & Kourtzi, 2002; Yovel & O’Toole, 2016).

Secondly, we justified the switch to US passports to reduce additional attentional demands and perceived salience brought about by an inconsistent ID template. Using a standardized ID template with blurred typeface implicitly encourages participants to ignore any irrelevant details (e.g., expiration date, name, birthdate; see Goal 1 of document authentication as described in Introduction) and instead devote attention to facial comparison (e.g., comparisons of target video and ID image; see Goal 2 of identity authentication as described in Introduction).

Our choice to diversify our professional pool was similarly motivated by both theoretical and applied rationales. At a theoretical level, we anticipated that different types of professional experience would involve a different *quantity* of experiences with fake identification cards. In line with logic laid out in Papesh and Goldinger (2014), we anticipated that bar door security professionals would likely encounter a higher percentage of fake IDs (in terms of both fraudulent documents and/or fraudulent identities) than access security professionals. If fake IDs are more prevalent in their occupational experiences, bar door security may be less susceptible to mismatch LPE errors. At an applied level, we also anticipated that different types of professional experience would also involve a different *quality* of experiences with identification checking more generally. Although much more challenging to typify, we anticipated these two types of professionals complete their identification checking tasks in, perhaps, meaningfully different ways.

## Experiment 2

Experiment 2 compared performance of non-professionals, bar door security professionals, and access security professionals on 100 identification trials between a target video and a US passport. We predicted that if the relative percentage of fraudulent ID cards in their professional experience conferred an advantage, then bar door security professionals (but not non-professionals or access security professionals) would commit fewer mismatch LPE errors. However, if these professional experiences did not transfer to this identification task, then we would predict no significant differences between professionals of either type and non-professionals.

### Method

#### Participants

All *N* = 714 participants were recruited through a Qualtrics panel (www.Qualtrics.com). Non-Professionals (*N* = 441; *M*age = 45.13 years, SD = 11.39; 42 chose not to answer; 282 females) participated in exchange for $6. Professionals were further divided into two groups. Bar security (i.e., panel respondents who self-reported as being currently employed as a bouncer or bartender, responsible for checking IDs as a routine part of their daily duties, with at least one year of professional experience; *N* = 138; *M*_age_ = 37.18 years, SD = 10.29; 14 chose not to answer; 75 females) participated in exchange for $20. Access security (i.e., panel respondents who self-reported as being currently employed as a border patrol officer, transportation security officer, police officer, or security guard, responsible for checking IDs a routine part of their daily duties, with at least one year of professional experience; *N* = 135; *M*age = 40.96 years, SD = 10.33; 15 chose not to answer; 56 females) participated in exchange for $30. All groups represented a diverse sample of participants (75.1% White/Caucasian, 4.5% Hispanic/Latinx, 9.1% Black/African-American, 4.8% Asian/Pacific Islander, 4.8% Multiracial, and 1.1% other; 4 participants chose not to answer).

Qualtrics panels operate online, and therefore we wanted to be assured that participant data were not contaminated by error variance not associated with the task at hand. Subsequently, *n* = 10 participants were excluded for failing to comply with instructions (e.g., answering “yes” on all image pairs, taking hours to complete a single trial). Therefore, the final sample included *n* = 704 participants.

#### Power analysis

Our decision to substantially increase our sample size, despite previous power analyses that suggested a smaller minimum *n*, was primarily guided by two factors. First, we acknowledged that several studies in the literature (e.g., White et al., 2014) have found no demonstrable difference between professionals and non-professionals on face matching tasks. However, many studies have adopted very conservative approaches to the minimum necessary sample size to capture the effect. As such, we wanted to err on the side of caution. Second, this video task combined with a new type of feedback (described in Methods below) is a newly adopted paradigm that has not been previously tested on any samples—students, professionals, or otherwise. Therefore, we wanted a large enough sample to capture any differing effects of this format, supposing that those effects exist.

We used G*Power (Faul et al., 2007) to perform an a priori power analysis for “ANOVA: Fixed effect, special, main effects and interactions” using a medium effect size *η*^2^ *p* = 0.09, which equates to *f* = 0.314. With power set to 0.90 and a numerator *df* of 2, the calculations suggested a minimum sample size of *n* = 132. With this value in mind, each group (i.e., non-professional, bar security, and access security) meet this minimum threshold to reveal an effect if it exists.

#### Materials

##### Identity matching task

Participants viewed the same 100 identities from Experiment 1. We replaced the static image with a moving target video presented beside a static image embedded in a US passport template with Adobe Photoshop on a black background (see Fig. 1). All videos and images were taken in a controlled environment. The target video was taken in front of a blue background in which the person turned their head from side to side for between 10 and 14 s. The video played continuously in a looping fashion until the participant made a decision. The US passport ID image was taken in front of a white background, and the passport template remained unchanged from trial to trial. The video and image were taken with different cameras, in different settings, between six months to five years apart.

#### Design and procedure

As in Experiment 1, all participants accessed the ID matching task and provided informed consent online through Qualtrics. All procedures were identical to Experiment 1, except that we replaced error-only feedback with visual repeat feedback (justified and more elaborately discussed in Experiment 2 Discussion). After making each decision, participants in the visual repeat feedback condition reviewed the trial and were asked to consider the similarities (on match trials) or differences (on mismatch trials) between the target identity and ID card identity before advancing to the next trial.

## Results

Replicating the pattern of results from Experiment 1, we found no reliable differences between professionals and non-professionals: Both groups exhibited the LPE as evidenced by differences in discriminability and criterion in the low prevalence conditions.

### Analysis of discriminability and response criterion

**Fig. 3:**
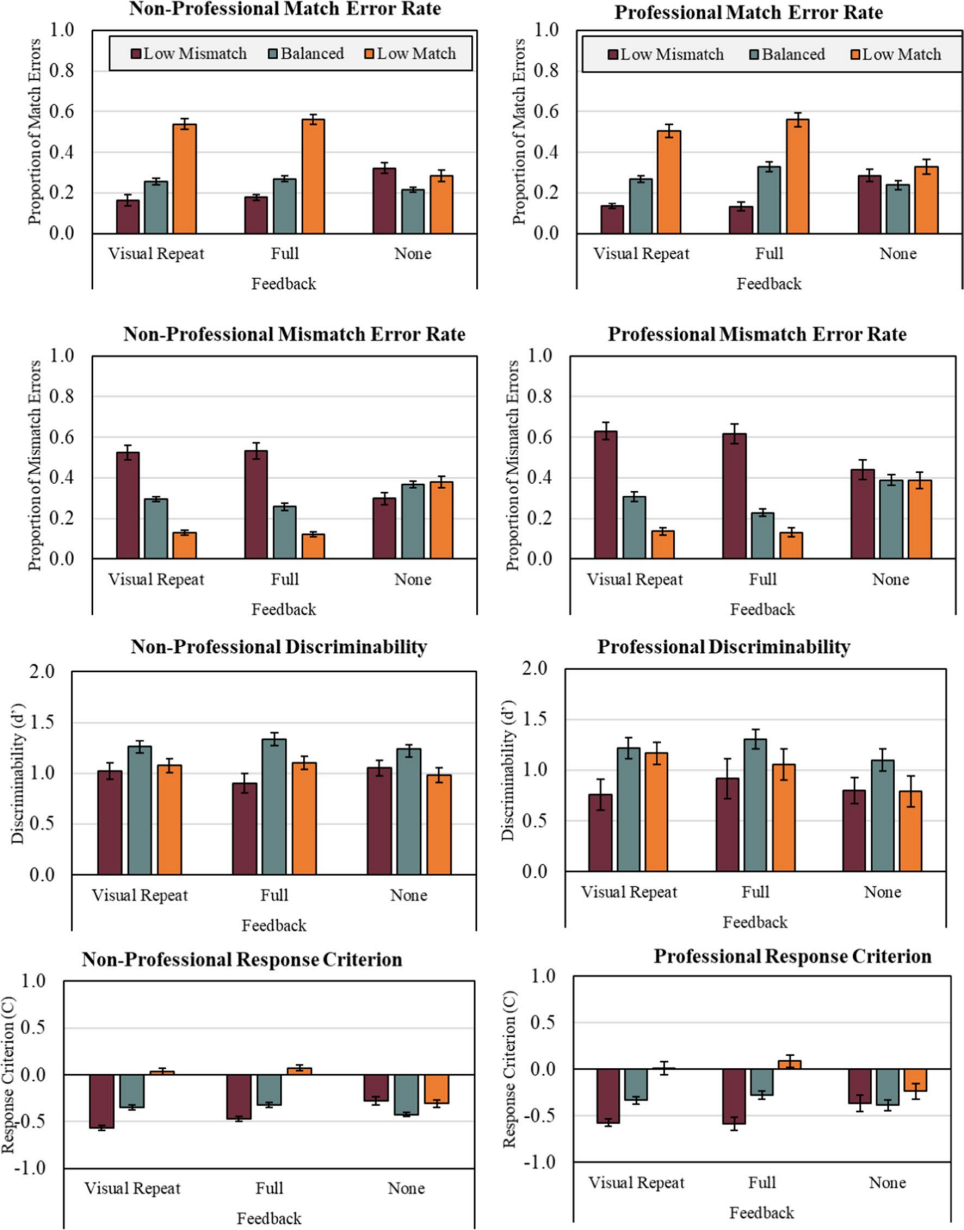
Average non-professional (left column) and professional (right column) match error rate, mismatch error rate, discriminability, and response criterion at each level of prevalence and feedback from experiment 2. Error bars represent standard error of the mean (SE)

As with Experiment 1, we again began with calculating and depicting match and mismatch error rates (see Fig. 3). Afterward, we conducted a 3 (Prevalence: Low Mismatch, Balanced, Low Match) × 3 (Feedback: None, Visual Repeat, Full) × 3 (Group: Non-Professionals, Bar Security, Access Security) factorial analysis of variance on discriminability measure d'. As with Experiment 1, the factor of note is Prevalence, which significantly affected discriminability, *F*(2, 680) = 25.07, *p* < 0.001, *n*^2^*p* = 0.069. Tukey's Bonferroni-corrected HSD found that Balanced prevalence yielded greater discriminability than Low Match prevalence (*p* < 0.001) and Low Mismatch prevalence (*p* < 0.001), which were not significantly different from one another. Feedback also affected discriminability, *F*(2, 680) = 3.89, *p* = 0.02, *n*^2^*p* = 0.011, although Tukey's HSD comparisons found no differences between individual Feedback conditions. Finally, Group also affected discriminability, *F*(2, 680) = 3.19, *p* = 0.04, *n*^2^*p* = 0.009, although Tukey's HSD comparisons found no differences between individual levels of the Group factor. There were no significant interactions among these factors.

We next conducted a 3 (Prevalence: Low Mismatch, Balanced, Low Match) × 3 (Feedback: None, Visual Repeat, Full) × 3 (Group: Non-Professionals, Bar Security, or Access Security) factorial analysis of variance on response criterion measure C. As can be seen in Fig. 3, criterion shifted primarily due to Prevalence and Feedback independently as well as through the interaction of these two factors. Prevalence significantly affected criterion, *F*(2, 680) = 140.46, *p* < 0.001, *n*^2^*p* = 0.292. Tukey's Bonferroni-corrected HSD tests found significant differences among all three prevalence conditions (*p*'s < 0.001). Low Match prevalence yielded the highest criterion followed by Balanced prevalence and Low Mismatch prevalence. Feedback also significantly affected criterion, *F*(2, 680) = 5.18, *p* = 0.006, *n*^2^*p* = 0.015. Tukey's HSD tests found that No Feedback yielded a significantly lower criterion than Full or Visual Repeat Feedback (*p*'s < 0.002), but the latter were not different from one another at the adjusted alpha level. These effects were accompanied by a significant Prevalence × Feedback interaction, *F*(4, 680) = 23.89, *p* < 0.001, *n*^2^*p* = 0.123. Planned follow-up comparisons found this interaction was driven by significant univariate effects of Feedback at Low Mismatch [*F*(2, 680) = 17.19, *p* < 0.001, *n*^2^*p* = 0.048], Balanced [*F*(2, 680) = 4.83, *p* = 0.008, *n*^2^*p* = 0.014] and Low Match [*F*(2, 680) = 32.51, *p* < 0.001, *n*^2^*p* = 0.087] prevalence conditions.

## Discussion

Despite the intuition that different types of professional experiences may improve detection of fake IDs, our results fail to support that position. On the contrary, we found that all three groups (non-professionals, bar security professionals, and access security professionals) largely followed the same patterns. Again, general cognitive mechanisms that predict the LPE more adequately explained our data than alternatives that assume different degrees of expertise as a function of different professional roles. Of rather small consolation, the only difference that emerged between professional groups was in response to visual repeat feedback. Whereas the low mismatch prevalence bar security professionals performed significantly worse than non-professionals on the visual repeat feedback, access security professionals did not.

In a sense, the visual feedback condition might be argued to provide both feedback (by way of confirming or disconfirming the participant’s decision) and attentional training (by cuing attention to either similarities or differences after a participant’s decision; see also Papesh et al. 2018 for a similar “burst” approach for attentional reorientation). Emerging evidence in sustained attention (Rothlein et al., 2018) suggests that task-relevant goals extracted from the context (in this case, prevalence rates) may promote the formation of attentional templates to optimize task performance. In response, participants rely upon these templates to reduce the reaction time necessary to make correct decisions. When the base rate for matches and mismatches is equivalent, as would be the case in the balanced prevalence conditions, then biasing the attentional template toward either similarities or differences would not be advantageous. However, when base rates are imbalanced, then biasing the attentional template be beneficial (see also goal hierarchy; Kluger & DeNisi, 1996). This benefit, though, would be theoretically symmetrical—visual repeating should affect both low match and low mismatch prevalence. Given that our data do not support this theoretical position, we are hesitant to draw any firm conclusions without replication. As a result, we reserve a full conversation of these findings for the General Discussion.

Turning to our third and final experiment, we sought to replicate the LPE findings and more closely examine professional experience. We again adopted the coarse-grained categorical classification (as authenticated by Qualtrics panels) of non-professionals, bar security professionals, and access security professionals. However, we also included an additional Professional Identity Training Questionnaire (PITQ, details in Experiment 3 Methods section; see also, Appendix) following the identification task. This questionnaire allowed us to characterize security professionals beyond a simple job title and occupational category.

The PITQ includes questions about four major aspects of professional experience (1) quantity of identification matching experience (e.g., years in occupation, shift length, percentage of shift devoted to task), (2) quality of identification matching experience (e.g., use of digital aids, types of image or video comparisons), (3) identification matching training experience (e.g., length, description, practice sessions, feedback), and (4) attitudes toward training and feedback (e.g., viewed as beneficial, other qualitative details). We reasoned that this information would be informative at a descriptive level (i.e., fill gaps in general knowledge about these facets of job experience) and at an inferential level (i.e., test whether professional experience predicts identification-matching performance).

As discussed in Introduction, one of the major goals of both professional experience and training is to render unfamiliar face matching (with which we do not all have expertise; cf. super-recognizers; Ramon et al., 2019a, 2019b) less error-prone by making it more similar to familiar face matching. However, empirical support is mixed. Nevertheless, if positions are to be made that professionals *should* gain expertise through years of experience and training, we reasoned that both should manifest in the form of a positive relationship with match hits and a negative relationship with mismatch false alarms.

In order for years of experience to improve screeners’ ability to recognize fraudulent identities, and not just fraudulent documents, government agencies (e.g., Australian Government Department of Foreign Affairs, 2020; U.S. Department of State, 2020) have outlined criteria to lessen the deleterious effects of facial variation. For example, US Passport facial photographs must meet criteria regarding sufficiently high resolution and size, standard backgrounds and lighting, front-facing pose with squared shoulders, and no accessories that might obscure parts of the face (e.g., hats, eyeglasses). This standardization serves both goals of authenticating the document itself (such that unacceptable photographic variations indicate a possibly fraudulent document) and authenticating the person presenting it (such that differences between the photograph and the person are reduced). In line with the differing complexity of the two goals of identity screening, the standardized elements of an authentic ID are concrete. Documents either do or do not possess the security features. Facial comparison is more abstract; the same facial features can support both match and mismatch decisions across screeners. Therefore, many screeners may only improve their ability to detect fraudulent documents via professional experience, without appropriately considering and improving facial image comparison.

If a professional is trained, that training should include practice and feedback about performance on identifying *both* fraudulent documents and fraudulent identities. This type of training has been implicated as the mechanism by which facial examiners (i.e., a separate class of facial comparison professionals who have demonstrated expertise as a function of mentorship and a focus on exhaustive deliberation) show improved performance compared to novices (Phillips et al., 2018; Towler et al., 2017; White et al., 2015). Information about which types of professionals complete training, and how often, is limited and/or kept intentionally private.

Recent literature, on the other hand, shows that training sessions for facial reviewers are typically relatively short courses (i.e., ranging from 1 h to 5 days) that customarily include description of the standard elements of the ID document, a brief introduction to facial anatomy, and identity screening exercises (e.g., Towler et al., 2019).

Unfortunately, the vast majority of training regimens are not research-informed. To improve the detection of fraudulent identities, research suggests that training exercises should include a variety of facial images (e.g., Gentry & Bindemann, 2019) that deviate from the standardized documents used for ID cards. This image variety should represent sufficient *within-person* variability, representing differences in images of the same person, and *between-person* variability, representing differences in images of two different people. Training should promote high identity-screening accuracy, even when either (a) an individual’s visual appearance changes drastically (e.g., weight loss/gain, natural aging, cosmetic alterations) from their ID facial image over time (Burton, 2013; Burton et al., 2016; Menon et al., 2015b) or (b) an ID facial image looks just as similar to its owner as an imposter.

## Experiment 3

To test the possible influence of professional experiences, Experiment 3 examined the degree to which responses to survey items predicted (or failed to predit) facial image comparison accuracy.

### Method

#### Participants

All *N* = 1474 participants (none of whom participated in any of the previous experiments) were recruited through a Qualtrics panel (www.Qualtrics.com) using the same compensation rates and criteria as Experiment 2. The sample represented non-professionals (*N* = 949;*M*age = 49.07 years, SD = 14.81; 1 chose not to answer; 625 females), bar security (*N* = 208; *M*age = 36.95 years, SD = 10.23; 4 chose not to answer; 94 females), and access security (*N* = 317; *M*age = 41.67 years, SD = 11.50; 122 females). All groups represented a diverse sample of participants (76.6% White/Caucasian, 8.6% Black/African-American, 4.0% Hispanic/Latinx, 4.3% Asian/Pacific Islander, 4.3% Multiracial, and 1.3% other; 8 participants chose not to answer). Twelve participants were excluded from analysis for the same types of instructional compliance violations as described in Experiment 2. The final sample of *N* = 1462 was subjected to data analysis.

#### Power analysis

Again, we selected our appropriate sample size based on an a priori power analysis. As this final experiment aimed to reveal differences between two types of professional experience, and included non-professional members for comparison, we again saw reason to be conservative in our sampling approach. We supposed that the effect may be only small to medium between these two professional groups. Using a *n*^2^*p* of 0.05 and corresponding *f* = 0.23, we used G*Power (Faul et al., 2007) to calculate the minimum sample size to reveal an effect with power set to 0.90 and numerator *df* set to 1. Output parameters revealed a minimum sample size of *n* = 202. As our smallest professional group size was *n* = 208, all three samples were sufficiently large to reveal an effect if it exists.

#### Materials

##### Identity matching task

Experiment 3 used the same identities and materials as Experiment 2.

##### Professional identity training questionnaire (PITQ)

The 34-item survey (see Appendix) contained 4 sections: (1) professional background, (2) professional experience, (3) professional training, and (4) attitudes toward training. The survey included a mix of close-ended and open-ended questions using a response-contingent presentation sequence.

#### Design and procedure

As in Experiments 1 and 2, all participants accessed the survey and provided informed consent online through Qualtrics. All procedures were identical to Experiment 2, except we replaced visual repeat feedback with guided visual feedback. After making an incorrect decision, participants in the guided visual feedback condition reviewed the trial and were asked to consider the similarities (on match trials) or differences (on mismatch trials) between the target identity and ID card identity before advancing to the next trial. Unlike the visual repeat condition from Experiment 2, visually guided feedback participants would automatically advance to the next trial if they made correct identity decision. Upon completion of the 100 trials, participants then completed the PITQ, provided demographic information, were debriefed, and thanked for their time.

## Results

As in Experiments 1 and 2, professionals and non-professionals both exhibited the LPE demonstrated by differences in discriminability and criterion when mismatch trials were rare. The addition of the Professional Identity Training Questionnaire revealed preliminary evidence that professionals’ false alarm rates (i.e., tendency to respond “match” to mismatch trials) are driven up among those who normally use normally additional technological aids when making ID verification during their work.

### Analysis of discriminability and response criterion

**Fig. 4:**
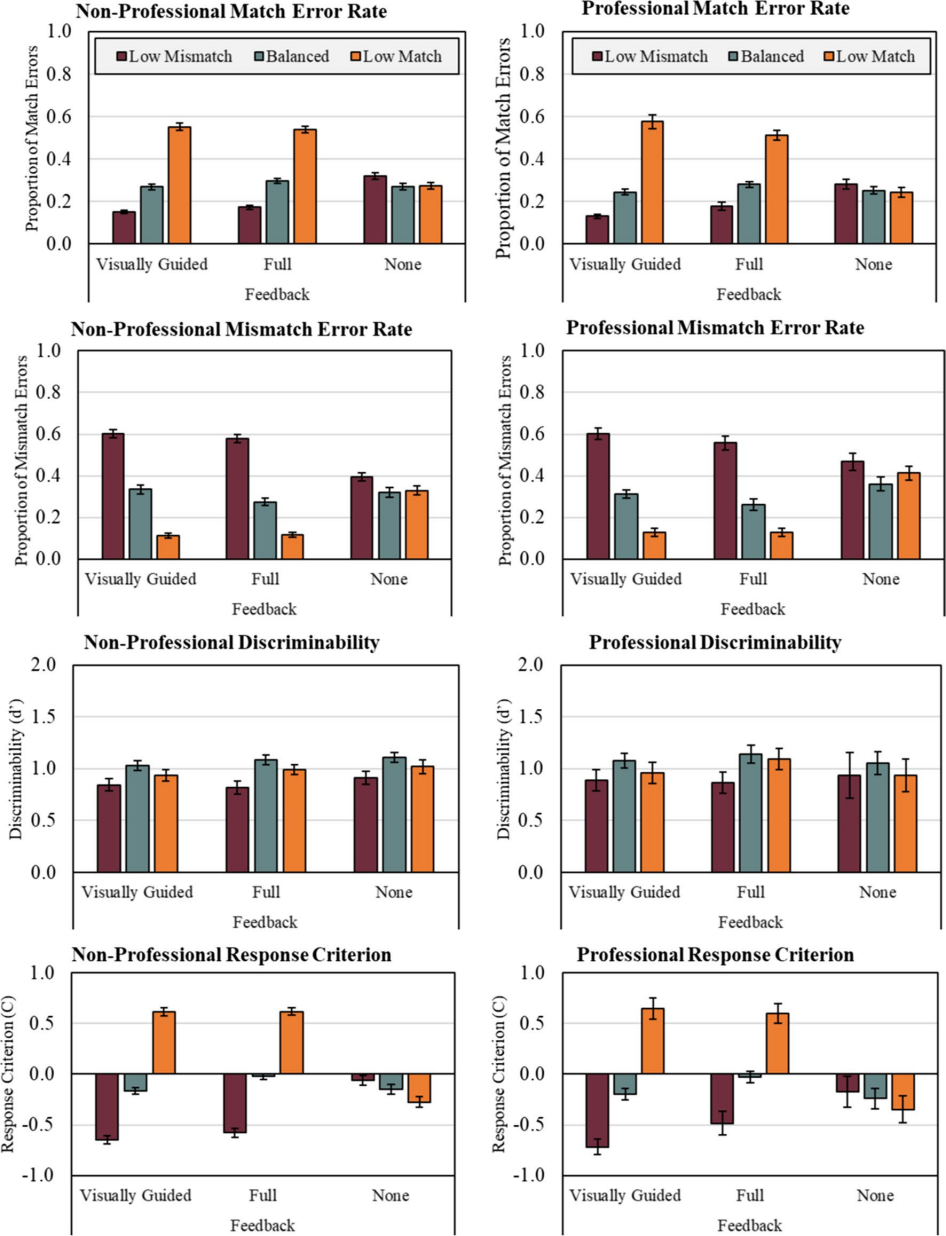
Average non-professional (left column) and professional (right column) match error rate, mismatch error rate, discriminability, and response criterion at each level of prevalence and feedback from experiment 3. Error bars represent standard error of the mean (SE)

As with Experiments 1 and 2, we again began by calculating match and mismatch error rates (see Fig. 3). Afterward, we conducted a 3 (Prevalence: Low Mismatch, Balanced, Low Match) × 3 (Feedback: None, Visually Guided, Full) × 3 (Group: Non-Professional, Bar Security, Access Security) factorial analysis of variance on discriminability measure d'. As can be seen in Fig. 4, only Prevalence significantly affected discriminability, *F*(2, 1425) = 9.14, *p* < 0.001, *n*^2^*p* = 0.013. Tukey's Bonferroni-corrected HSD found that Low Mismatch prevalence yielded poorer discriminability than Balanced (*p* < 0.001) and Low Match prevalence (*p* < 0.02), which were not significantly different from one another. No other factors yielded significant main effects or interactions.

We next conducted a 3 (Prevalence: Low Mismatch, Balanced, Low Match) × 3 (Feedback: None, Visually Guided, Full) × 3 (Group: Non-Professional, Bar Security, Access Security) factorial analysis of variance on response criterion measure C. As can be seen in Fig. 4, criterion shifted primarily due to Prevalence and Feedback independently as well as through the interaction of these two factors. Prevalence significantly affected criterion, *F*(2, 1425) = 169.03, *p* < 0.001, *n*^2^*p* = 0.192. Tukey's Bonferroni-corrected HSD found that Low Mismatch prevalence yielded a lower criterion than Balanced (*p* < 0.001) and Low Match prevalence (*p* < 0.001). In turn, Balanced prevalence yielded a lower criterion than Low Match prevalence (*p* < 0.001). Feedback also significantly affected criterion, *F* (2, 1425) = 19.62, *p* < 0.001, *n*^2^*p* = 0.027. Tukey's HSD tests found that No Feedback yielded a significantly lower criterion than Full or Visually Guided Feedback (*p*'s < 0.001), but the latter were not different from one another at the adjusted alpha level. These effects were accompanied by a significant Prevalence x Feedback interaction, *F*(4, 1425) = 67.91, *p* < 0.001, *n*^2^*p* = 0.160. Planned follow-up comparisons found this interaction was driven by significant univariate effects of Feedback at Low Mismatch [*F*(2, 1425) = 33.11, *p* < 0.001, *n*^2^*p* = 0.044], Balanced [*F*(2, 1425) = 6.46, *p* = 0.002, *n*^2^*p* = 0.009] and Low Match [*F*(2, 1425) = 116.57, *p* < 0.001, n *n*^2^*p* = 0.141] prevalence conditions.

#### Regression analyses on survey responses

One of the major goals of Experiment 3 was to determine whether aspects of professionals’ employment and personal histories predicted success at the ID screening task (see descriptive statistics, see Table 1). We carried out regression analyses on hits and false alarms between bar security and access security professionals. The four predictors entered into the models were number of days of pre-employment training, duration (in minutes) of a typical ID verification shift, duration (in years) of ID screening employment, and whether they use any type of computerized aid in their ID screening procedures. This last predictor was dummy-coded with 0 representing "no" responses and 1 representing "yes" responses. Only members of the professional sample who indicated that they screen IDs as part of their current job (*n* = 403) were included in these analyses^1^.

**Table 1:** Demographic characteristics and survey responses from security officers and bartenders in experiment 3

	Security	Bartender
Total	317	202
*Ethnicity*		
Black/African-American	26	24
Hispanic/Latino	11	14
White/Caucasian	248	137
Asian/Asian American/Pacific Islander	13	10
Other/chose not to respond	19	18
*Mean (SD) age*	41.74 (12.12)	36.94 (10.28)
*Self-reported gender*		
Female	123	109
Male	194	93
*Security field*		
Airport security	21	-
Border patrol officer	17	-
Police officer	120	-
Security guard	151	-
Other	7	-
*Years working*		
< 1	27	38
1 to 5	77	82
5 to 10	75	57
> 10	138	26
Mean (SD)	10.36 (8.83)	6.57 (6.67)
*Typical work shift length*		
Mean (SD)	9.10 (2.29)	8.19 (2.20)
Screens IDs as current job duty	239	164
Mean (SD) years in this position	8.17 (8.06)	5.27 (5.18)
Mean (SD) minutes screening IDs in work shift	212.06 (250.99)	252.56 (294.39)
Received training	83	52
Mean (SD) days training	8.75 (9.50)	2.18 (4.92)
Undergoes practice sessions	70	43
Receives feedback on ID screening	55	42
*ID screening media (select all that apply)*		
Person-to-photograph	217	140
Photograph-to-photograph	80	44
Video-to-person	70	24
Video-to-photograph	54	24
Person-to-person	64	21
Uses digital aids	35	27

“SD” refers to standard deviation of the mean

The first regression analysis examined hits. No predictors reached significance, but Years of employment marginally predicted match hit rate, b = − 0.003, SE = 0.002 *t*(402) = − 1.91, *p* = 0.06, 95% CI [− 0.005, 0.000]. Additional information can be found in Table 2.

**Table 2:** Multiple regression results for relevant survey predictors for committing hit and false alarm decisions in the ID matching task used in experiment 3

	*b*	SE *b*	*β*
*Outcome: match hits*			
Days of pre-employment ID-screening training	.00	.02	− .01
Years of ID-screening experience	.00	.00	− .10^†^
Minutes of ID-screening during typical work shift	.00	.00	− .03
Use of digital aids in screening (0 = No, 1 = Yes)	.03	.03	.05
*Outcome: mismatch false alarms*			
Days of ID-screening training	.02	.03	.03
Years of ID-screening experience	.00	.00	− .01
Minutes of ID-screening during typical shift	.00	.00	.02
Use of digital aids in screening (0 = No, 1 = Yes)	.12	.04	.17*

*Indicates significant prediction *p* < .05

^†^Indicates marginal prediction *p* < .06

**Fig. 5:**
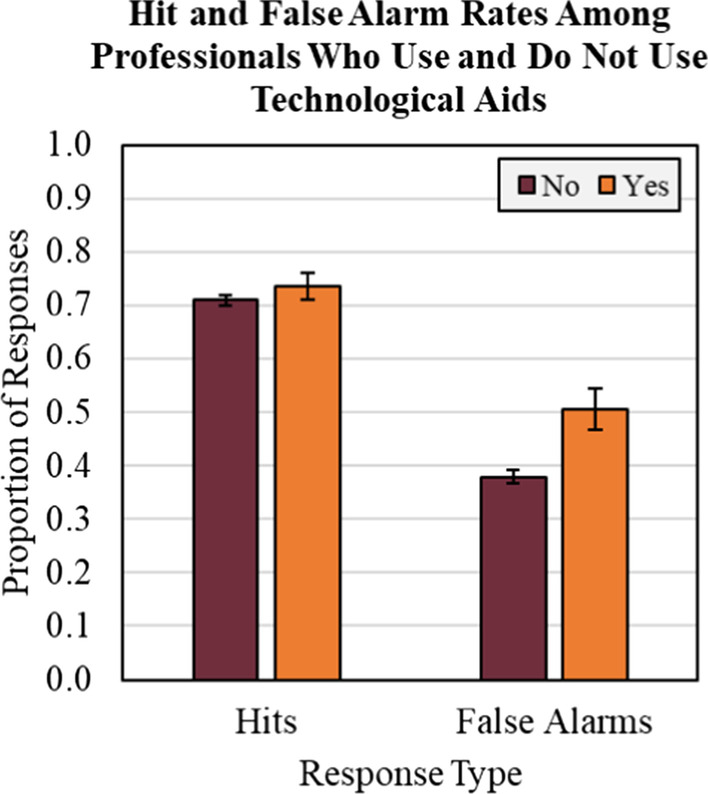
Average professional hit and false alarm rates among those who reported normally using technology and those who do not in experiment 3. Hits are derived from correct match trial responses, and false alarms are derived from incorrect mismatch trial responses. Data are collapsed across prevalence and feedback conditions. Error bars represent standard error of the mean (SE)

The second regression analysis examined false alarms to mismatches. Use of digital aids in ID screening positively predicted mismatch false alarms, *b* = 0.118, SE = 0.037 *t*(402) = 3.22, *p* = 0.001, 95% CI [0.046, 0.190], meaning that those professionals who use some kind of computerized assistance when checking IDs on the job were more likely to commit errors in our mismatch trials (see Fig. 5). No other predictors reached significance.

## Discussion

Once again, professional screening experience did not improve identity-matching performance. Instead, Experiment 3 largely replicated the pattern of the two previous studies: professionals commit the LPE at similar rates as non-professionals. We more closely examined nuance among facial reviewers in terms of training, typical identification matching duties, and digital additions (e.g., technological aids). Despite our attempts to more clearly define the roles and relationship between professional experience and the LPE, none of the following questionnaire components predicted improvements in overall performance or mismatch LPE errors. To be fair, years of experience suggested moderate prediction of match performance, but that relationship was weak and perhaps unreliable given the large sample size. We would need to replicate this finding at a more stringent alpha level in order to have more trust in its reliability.

The large sample size, on the other hand, did allow for more variability in responses to the PITQ. Using a close-ended and open-ended approach, participants provided both presence/absence responses (e.g., did you experience any professional identity training?) as well as follow-up details (e.g., if yes, please explain). The quantitative responses allowed for our inferential analysis above, but the qualitative results may also be seen as informative (for access to all responses, see OSF website listed below).

The only question that predicted mismatch errors was the use of digital aids: professionals who reported using them actually produced *more* mismatch errors than those who did not (or non-professionals). This finding is striking and worthy of additional consideration. Among those who thoroughly described these tools, reports varied from tools for verifying authenticity of documents (e.g., watermarks, special inks, etc.) to automatic facial matching against mugshot databases. We hesitate to make claims about specific tools, as our experiment was not devised to systematically test the effects of specific pieces of software. Nonetheless, to the extent that these digital aids are designed to improve identification-matching performance, our results suggest that such resources could be more wisely invested in other professional improvement approaches that benefit public safety. We explore possible mechanisms behind this result and recommendations in the General Discussion.

## General discussion

Across three studies, we more thoroughly investigated the role of professional experience in an ID screening task comprised of different ratios of matched and mismatched face pairs. In Experiment 1, we replicated the findings of Weatherford et al. (2020; Experiment 2) using static stimuli and US driver licenses. We incorporated feedback as a possible intervention: full feedback after every trial, feedback only after error trials, or no feedback. In Experiment 2, we more strongly approximated a real-world context by including video targets compared with standardized US passports. We also incorporated forms of visual feedback that were designed to reduce the LPE in all samples (professional and otherwise) by disrupting attentional templates and criterion shifting that drive the LPE. Further, we subdivided types of professional experience into two broad categories: bar security and access security. Finally, Experiment 3 adopted the same video target paradigm as Experiment 2 and introduced a response-contingent visual feedback condition (as opposed to the visual repeat condition in E2). Most importantly, we integrated a professional identity training questionnaire (PITQ) to more closely consider the relationship between task performance and different facets of professional experience.

All three studies supported the same broad outcomes: 1) professionals suffer from LPE mismatch errors to the same extent as non-trained members of the general public, 2) various forms of feedback and corrective intervention do not reliably ameliorate the LPE, and 3) self- reported differences in professional experience and training do not confer the expertise necessary to combat this problem, and may actually deteriorate performance in some cases.

On the face of it, these results may seem disheartening to both face researchers and security practitioners. However, closer analysis of these findings allows us to draw two firm conclusions that warrant additional consideration. First, the failure to identify low prevalence fake IDs represents a serious real-world problem that may compromise job performance and public safety. Second, the nature of professional experience (Balsdon et al., 2018; Towler et al., 2018, 2019) can offer key insights into the important role of empirically informed training and mentorship to promote improvements in facial identification performance that combat the LPE.

### Fake IDs pose a serious security threat

To the extent that cognitive biases revealed by basic research findings apply to the real world, the LPE represents a serious problem for ID verification. By all estimates, imposters rarely present fraudulent documents at security checkpoints. If human screeners are not adequately prepared to detect imposters, then researchers need to partner with practitioners to address this problem.

As an attempt to increase the efficacy of job performance, many professionals use digital aids. Several currently adopted technologies credit earlier military technology, such as the Biometric Automated Toolset (BAT) and Handheld Interagency Identity Detection Equipment (HIIDE; Xml et al., 2007). These tools assume that routine checks of individuals need only involve the human screener. However, under conditions when a database of biometric information suggests additional scrutiny, individuals under suspicion of criminal activity may be flagged on the basis of fingerprints, iris scans, facial photographs, gait patterns, and/or biographical information that matches established databases. In order for these tools to be effective, human screeners must have consistent access to the technology and databases must be constantly updated (a standard that would be difficult for any agency to meet).

Experiment 3 found that professionals who indicated use of digital aids tended to commit more mismatch errors than professionals who did not. These digital aids, and the extent to which they are used, varied among respondents. Therefore, we hesitate to draw firm conclusions about any specific tools used by professional security personnel.^4^ However, research elsewhere in applied cognition has found that even pristinely accurate digital tools can disrupt user cognition. For example, use of global positioning software can disrupt participants’ cognitive map formation, leading to poorer wayfinding later without such software compared to those who learned topographies through experience alone (Ishikawa et al., 2008; Minaei, 2014). Both this set of findings and our own imply that domain-specific digital tools may aid those using them in the moment, but may deleteriously affect learning.

Future research should explore the magnitudes of these tools’ various effects on identity matching ability. For other visual search tasks, such as baggage screening (e.g., Huegli et al., 2020), automation might improve screener performance. However, visually searching facial cues is fundamentally different than visually searching object cues (e.g., Curby & Gauthier, 2010; McKone et al., 2007). Computer aids may yield a higher successful detection rate for concrete aspects of the document itself; however, human screeners still outperform many algorithms on more nebulous visual cues associated with identity (e.g., Phillips & O’Toole, 2014). Digital aids may focus attentional resources on identifying fraudulent documents (e.g., black light scanners) at the expense of identifying fraudulent identities. To the extent that an ID is judged as an authentic document by the digital aid, experiments could explore whether confirmation bias (e.g., Rajsic et al., 2015) guides visual search by attenuating goal-directed attention after the first goal is satisfied (see also, multiple target search satisfaction; Adamo et al., 2019; Biggs & Mitroff, 2014; Cain & Mitroff, 2013; Thornton & Gilden, 2007). Although criterion shifting is a well-known source of LPE errors in facial comparison tasks, these real-world possibilities may also lend support to additional errors driven by early search termination.

One justifiable recruiting expense may be to solicit assistance from individuals with superior facial recognition skills (e.g., super-recognizers; SRs; Davis et al., 2016; Ramon et al., 2019a, 2019b; Russell et al., 2009). Studies show that super recognizers may not be fully aware of their skills (i.e., limited metacognitive awareness; e.g., Bate & Dudfield, 2019) and fail to adequately calibrate their confidence in response to face-matching decisions (e.g., Gettleman et al., 2020; Ramon et al., 2019a). Even more challenging, SRs might not take interest in security screening, regardless of their enhanced abilities. Therefore, recruiting identity screeners in professional circles may be problematic. Nonetheless, working with super recognizers offers a potentially promising way forward, as these individuals are more successful at recognizing faces despite common sources of within-person variability such as age gaps, day-to-day changes in facial hair or makeup, and suboptimal viewing conditions (Bate, Frowd, et al., 2019b; Ramon et al., 2019a; Stacchi et al., 2020; although SRs still suffer from Own Race Bias; Bate, Bennetts, et al., 2019a). These challenges to face matching are typical for professional screeners, given that many identification documents (e.g., US Passports) are valid for up to ten years, during which time any bearer of an ID would undergo some substantive visual changes. To the extent that these types of skills can be trained, the most advantageous role for SRs might be to inform facial comparison training.

### Feedback contributes to low prevalence errors

As a general pattern, our data (see also, Papesh et al., 2018) do not support the use of feedback as an intervention to combat the LPE. When match and mismatch trials were imbalanced, participants’ criterion shifted with explicit feedback (see Experiment 3 data for strongest evidence of the crossover interaction). In a sense, this finding is ideal because trial-by-trial feedback would be impossible in real-world security settings. If the “ground truth” were known about the accuracy of a human screener’s decision, there would be no need for the human screener.

Interestingly, this pattern is not unique to ID matching tasks. Coining the term Prevalence-Induced Concept Change (PICC), Levari et al. (2018) reported data that might shed light on the theoretical mechanisms driving the differences. PICC posits that low prevalence prompts observers use their own implicit feedback (on the basis of their own imbalanced responding) to expand the boundaries of an ambiguous concept (e.g., color labels across a spectrum, facial expressions that signal threat) by shifting their criterion to consider *more* stimuli as exemplars of an infrequent category. For instance, after establishing a balanced prevalence between colored dots ranging from “mostly blue” to “mostly purple,” participants were more likely to miscategorize colors in the purple range as blue when blue stimuli became infrequent. If training regimens could successfully establish and maintain an expectation for balanced prevalence rates between authentic and fake IDs (or integrate high-prevalence bursts, e.g., Papesh et al., 2018), human screeners might prefer committing a false negative (i.e., rejecting an authentic ID) over a false positive (i.e., accepting a fake ID). However, more research would need to test this possibility before making training recommendations.

### Training to combat this problem needs to be intentional and research-informed

As corroborated by our PITQ findings, many employers invest substantial resources to improve facial reviewers’ performance on their routine screening tasks. While research-informed training is showing early success for other professional search domains within airport security (Biggs et al., 2018; e.g., Biggs & Mitroff, 2019; Swann et al., 2019), findings within facial comparison screening remain mixed (e.g., Towler et al., 2019). Outside of training that focuses on authenticating documents, which may lead to confirmation bias issues, any successful training regimen *must* meaningfully nurture a screener’s ability to differentiate *within-person* variability under circumstances when someone presents a genuine ID from *between-person* variability when someone presents an imposter ID. In order for training and on-the-job experience to promote facial comparison expertise (White et al., 2015), the training needs to include (a) images with facial variability (e.g., Kramer & Ritchie, 2016; Matthews & Mondloch, 2018; Menon et al., 2015a) such as the Good, the Bad, and the Ugly facial training set (Phillips et al., 2012), (b) guided mentorship (see FISWG, 2011 criteria for facial examiners), and (c) an emphasis on accuracy over speed.

Relatedly, research needs to more strongly examine how training and professional experience transfer to job performance. Despite extensive previous experience with imbalanced ratios of authentic to fraudulent ID cards, our professionals did not apply of their knowledge of real-world base rates to the experimental task (which would have otherwise made their performance *worse* than non-professionals). One possibility is that professionals may compartmentalize the experimental/training scenario as related, but distinct, from the actual screening task itself. If that lack of transfer can account for our findings, future research should explore how training performance actually predicts real-world performance before making specific recommendations.

## Conclusions

To conclude, our results reveal that professionals and non-professionals do not differ in their ID matching abilities. When professionals make errors, it is likely due to limited metacognitive awareness of their error rates and their possible reliance upon digital aids, which is especially pronounced when we only examine low prevalence conditions. Future research should explore the extent to which professional screeners may merely invest their resources to verifying the authenticity of the document itself rather than the identity, and how they calibrate their confidence in their identity matching decisions.

### Supplementary Information

**Additional file 1.** **Supplemental Figures:** Receiver Operating Characteristic (ROC) curves by prevalence, feedback, and professional status. Areas under the curve (AUC) represent total area. Partial area under the curve (pAUC) represent overlapping regions shared along the x axis by two groups under comparison.

## Data Availability

The experimental programming and datasets used and/or analyzed during the current studies are available from the corresponding author at https://osf.io/unj9t/. Stimuli are available by reaching out to the corresponding author at dawn.weatherford@tamusa.edu.

## Abbreviations

AUC: Area under the curve
FISWG: Facial Identification Scientific Working Group
LPE: Low prevalence effect
pAUC: Partial area under the curve
PICC: Prevalence-induced concept change
ROC: Receiver operating characteristic
SR: Super recognizer

## Footnotes

1 Our disparate compensation rates throughout all three experiments represent an increased incentive for our professional samples, which might have otherwise been more challenging to recruit. Increased compensation is standard practice across many fields who recruit professionals, and we do not see any evidence to suggest that it altered participants’ patterns of responding.

2 We recruited our sample to approximate the racial/ethnic and gender demographics of the 2010 US Census. Participants were randomly assigned to conditions such that aggregate values across all conditions mirror demographic characteristics within conditions.

3 For a more nuanced treatment of the data that does not collapse across degrees of certainty reflected in participants’ interpretation of differences between Definitely, Probably, and Maybe, we also created receiver operating characteristics (ROC) curves. This approach allowed us to compare partial area under the curve (pAUC) between conditions. The overall pattern of results was similar to the signal detection measures reported above. Therefore, we direct the interested reader to our Additional file 1 for the analysis and report of those data.

4 Tools described by respondents varied in function from document verification (i.e., determining whether the identification document is a legitimately government-issued) to facial identity verification aids.

## Declarations

### Significance statement

In response to various worldwide security concerns at borders and other ports of entry, professional screeners commonly restrict access of goods and services to authorized individuals who present an authentic ID card. For example, a potential traveler may produce stolen, borrowed, or inauthentic passport documents. Professional screeners need to maintain safety by identifying such imposters, while still allowing lawful passengers through. Although technological advancements such as automatic face recognition systems and various methods of biometric scanning may seem like attractive alternatives to replace human screeners, such technologies face many of the same challenges as human recognizers, while also raising concerns about ethics, transparency, and accountability. Therefore, the bulk of imposter detection duties has been and is being performed by humans. Subsequently, our experiments explore cognitive mechanisms that may underlie ID matching task performance with aims to support data-driven recommendations for pre-employment screening, policies and procedures, and job-related training for security professionals. Best practices and appropriate interventions are important to ensure stakeholder safety at security checkpoints where screening IDs is common and necessary.
