# Supplementary material for: When experience does not promote expertise: security professionals fail to detect low prevalence fake IDs
Source: Cogn Res Princ Implic. 2021 Apr 1;6:25. doi: 10.1186/s41235-021-00288-z (PMC8017042; doi:10.1186/s41235-021-00288-z)
Supplement: Supplementary file 1 — Additional file 1. Supplemental Figures: Receiver Operating Characteristic (ROC) curves by prevalence, feedback, and professional status. Areas under the curve (AUC) represent total area. Partial area under the curve (pAUC) represent overlapping regions shared along the x axis by two groups under comparison. [file 41235_2021_288_MOESM1_ESM.docx]

**Supplementary Material: Partial Area under the Curve (pAUC) Results**

**Experiment 1**

An additional way to consider the current data is with partial area under the curve (pAUC) analysis, which allows us to compare differential effects of Prevalence on discriminability at different criterion levels. Additionally, it allows us to consider all possible decisions (i.e., 1-6 instead of collapsed into yes/no). As described in the Methods above, participants used values “1”, “2”, or “3” to indicate that faces are not the same identity, and “4”, “5”, and “6” to indicate that faces are the same identity. The cumulative proportions of “1”,“2”, “3”, “4”, “5”, and “6” responses made by each participant within each Prevalence and Feedback combination from 6 (the highest criterion level) to 2 (the lowest criterion level) were calculated for each matches and mismatches, and plotted in ROC space for three curves representing Prevalence in each Feedback condition (see Figure 2). The space is arranged such that accuracy for match trials is plotted along the vertical axis ranging from 0 to 1 and mismatch trials plotted along the horizontal axis from 0 to1. The diagonal from coordinate 0,0 to 1,1 indicates chance performance, with coordinates above indicating accuracy above chance and coordinates below indicating accuracy below chance. Because our curves do not span the entire hypothetical distribution of response values from 0,0 to 1,1 in ROC space, analyses were conducted on the truncated portions of curves where conditions being compared overlap. We used the pROC toolbox for R to compute these analyses (Robin et al., 2011).

Our first interest was whether prevalence affected accuracy as displayed in a receiver operating characteristic (ROC) space ([Supplementary](#_bookmark1) Figure 1), collapsed across all other variables. This analysis would establish whether our experiment replicated the Low Prevalence Effect (LPE). Our second interest was to examine whether professional ID screeners were as susceptible to committing LPE errors as nonprofessionals in our sample. For each analysis, we computed partial area under the curve (pAUC) scores for each ROC curve and conducted pairwise comparisons among the conditions of interest (see Table 1 for collapsed analyses and Table 2 for professional and non- professional screening data).

**Prevalence Collapsed across All Other Variables.** We adjusted the alpha level for significance decisions to .017 to account for the following three analyses: low mismatch prevalence vs. balanced mismatch prevalence, low mismatch prevalence vs. low match prevalence, and balanced mismatch prevalence vs. low match prevalence. For the comparison between low mismatch prevalence and balanced prevalence, the area spanned by the low condition (pAUC = .43) was less than the area spanned by the balanced condition (pAUC = .52), D = 4.57, *p*< .001. For the balanced and low match conditions, the area spanned by balanced prevalence (pAUC = .37) was greater than low match prevalence (pAUC = .31), D = 3.42, *p* < .001. Comparisons between the low mismatch and low match conditions yielded no differences in pAUCs.

**Professionals and Non-professionals Under Low Prevalence.** Comparisons between professional and non-professional samples showed no differences in pAUCs.


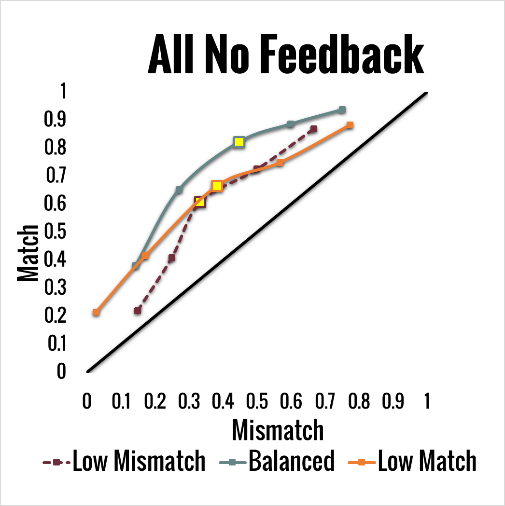

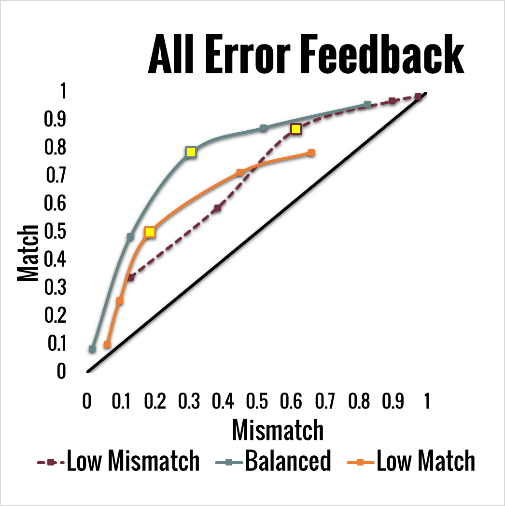

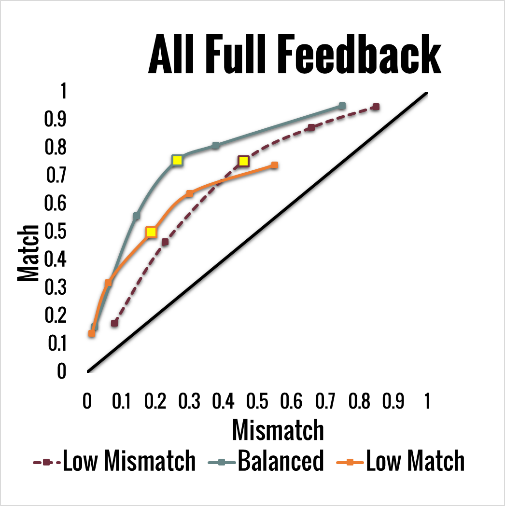


***Supplementary Figure 1.*** Receiver Operating Characteristic (ROC) Curves by prevalence group. To ease comparison, the third point on each ROC for low mismatch, balanced, and low match prevalence is highlighted to show shift along the x-axis.

**Experiment 2**

**Prevalence Collapsed Across all Other Variables.** As in Experiment 1, we first analyzed prevalence conditions only (for group data, see [Figure 3](#_bookmark2)). For the comparison between low mismatch prevalence and balanced prevalence, the area spanned by low mismatch prevalence (pAUC = .45) was less than the area for balanced prevalence (pAUC = .50), D = 9.34, *p* < .001. For the balanced prevalence to low match prevalence comparison, balanced prevalence (pAUC = .36) spanned a smaller area than low match prevalence (AUC = .32), D = 8.28, *p* < .001. Low mismatch and low match conditions were equivalent, *p* > .2.

**Professionals and Non-professionals Under Low Prevalence.** This analysis examines differences among Bar security professionals, Access security professionals, and Non- professionals under Low prevalence. For the comparison between Bar security professionals and Non-professionals, the area spanned by Bar security (pAUC = .52) was less than the area spanned by Non-professionals (pAUC = .57), D = 3.29, *p* = .001. Remaining comparisons showed no differences in pAUC scores, *p*s > .10.


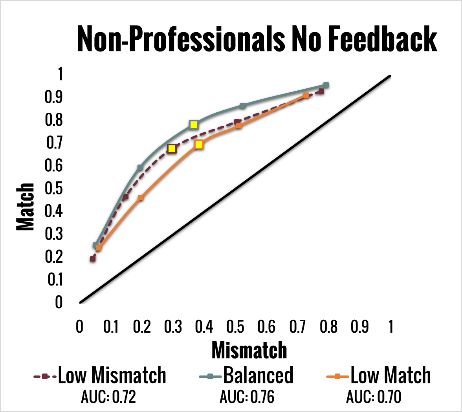

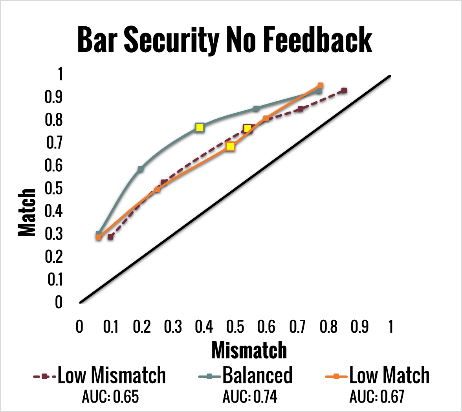

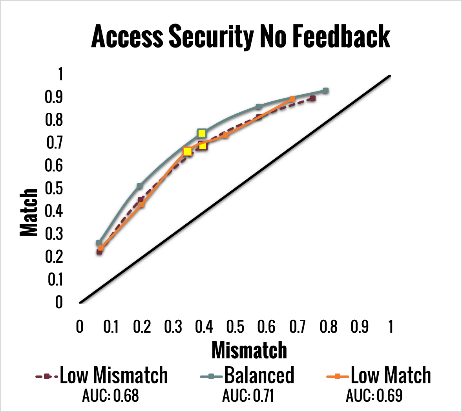


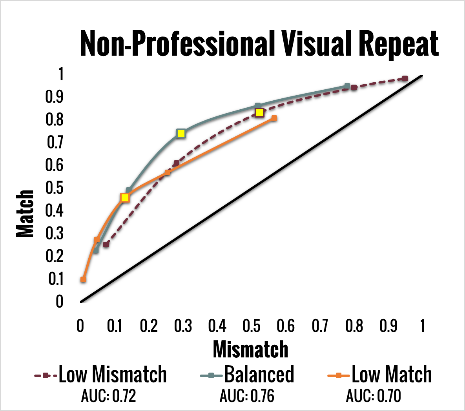

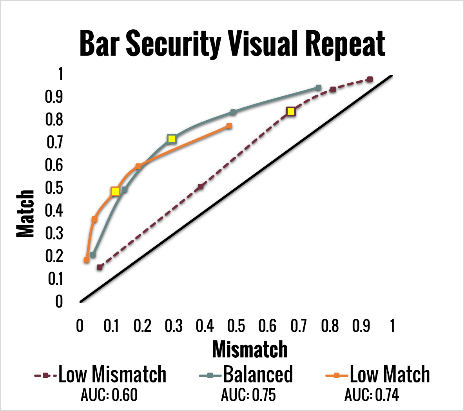

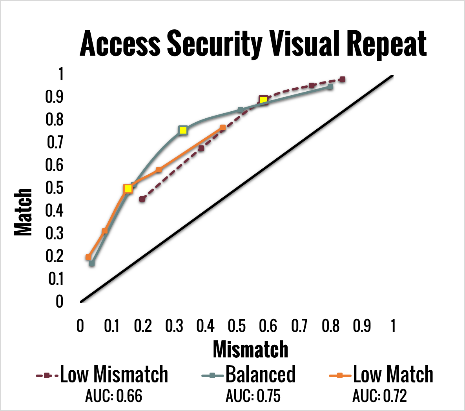


***
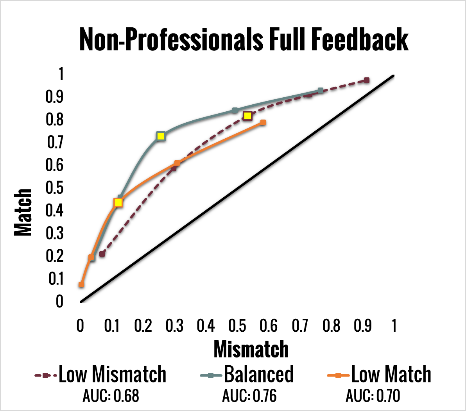

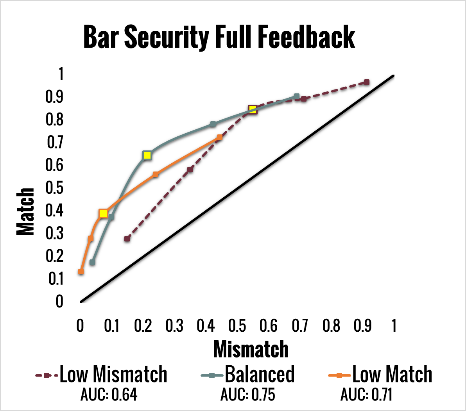

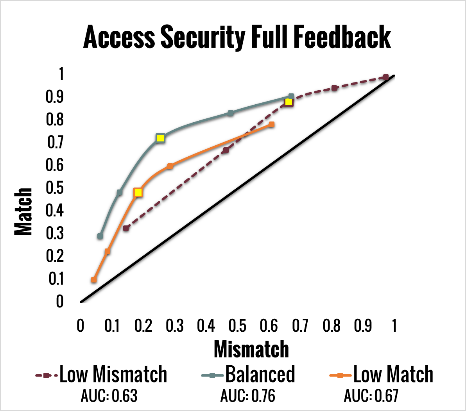
***

***Supplementary Figure 2*.** Receiver Operating Characteristic (ROC) Curves by prevalence group for the no feedback (a), full feedback (b), and visual repeat (c) conditions in Experiment 2. Area Under the Curve (AUC) calculated using the trapezoidal method. To ease comparison, the third point on each ROC is highlighted. Although the visual repeat and full feedback conditions show a more robust shift along the x-axis, differences in the no feedback conditions were mixed.

**Experiment 3**

**Prevalence Collapsed Across all Other Variables.** As in Experiment 1, we first analyzed prevalence conditions only (for group data, see [Figure 3](#_bookmark2)). For the comparison between low mismatch prevalence and balanced prevalence, the area spanned by low mismatch prevalence (pAUC = .45) was less than the area for balanced prevalence (pAUC = .50), D = 9.34, *p* < .001. For the balanced prevalence to low match prevalence comparison, balanced prevalence (pAUC = .36) spanned a smaller area than low match prevalence (AUC = .32), D = 8.28, *p* < .001. Low mismatch and low match conditions were equivalent, *p* > .2.

**Professionals and Non-professionals Under Low Prevalence.** This analysis examines differences among Bar security professionals, Access security professionals, and Non- professionals under Low prevalence. For the comparison between Bar security professionals and Non-professionals, the area spanned by Bar security (pAUC = .52) was less than the area spanned by Non-professionals (pAUC = .57), D = 3.29, *p* = .001. Remaining comparisons showed no differences in pAUC scores, *p*s > .10.


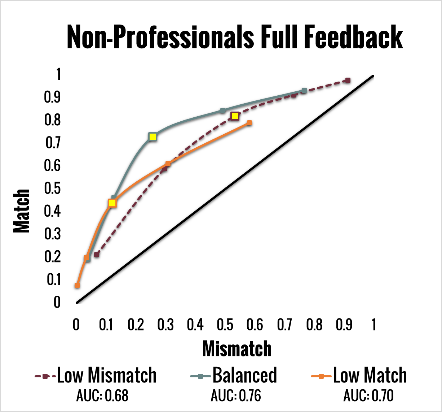

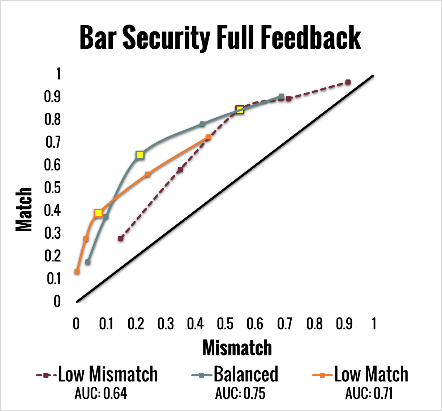

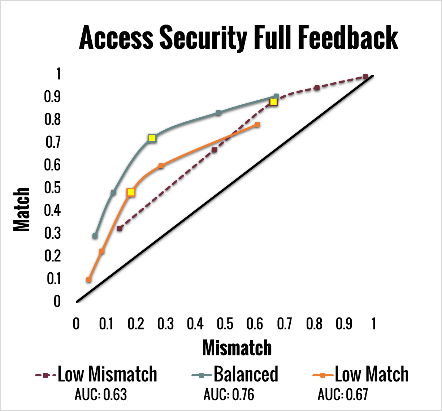


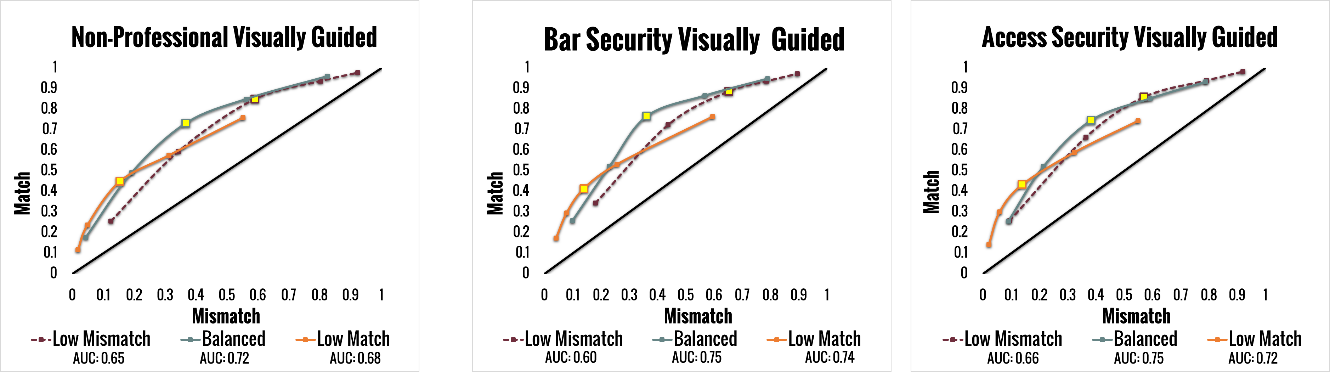

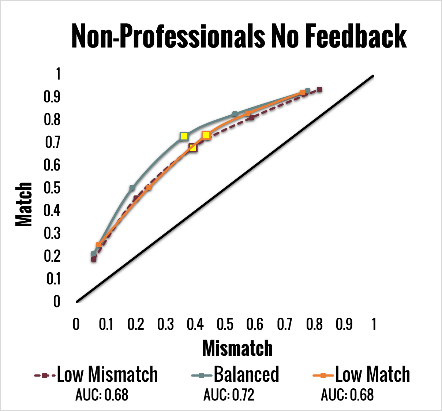

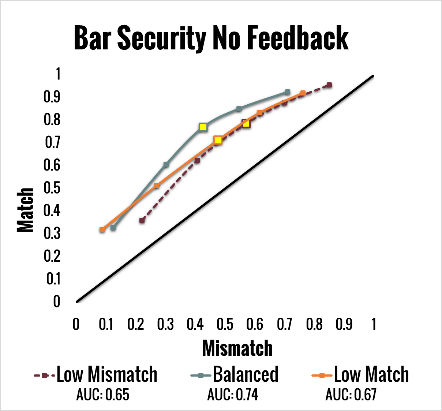

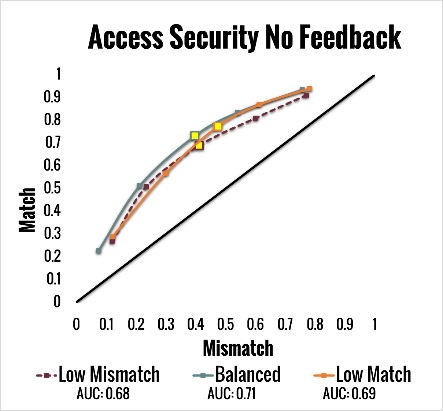


***Supplemental Figure 3***. Receiver Operating Characteristic (ROC) Curves by prevalence group for the no feedback (a), full feedback (b), and visual repeat (c) conditions in Experiment 3. Area Under the Curve (AUC) calculated using the trapezoidal method. To ease comparison, the third point on each ROC is highlighted. Although the visual repeat and full feedback conditions show a more robust shift along the x-axis, differences in the no feedback conditions were mixed.

**Supplementary Table 1.** Lower and upper ROC curve overlap boundaries used for each partial area under the curve (pAUC) analysis of prevalence collapsed across all other variables, including D value for each comparison (*α* = .017).

| Partial ROC Boundaries | | | |
| --- | --- | --- | --- |
| Comparisons | Lower | Upper | D |
| Experiment 1 |  |  |  |
| Low Mismatch vs. Balanced | .22 | .88 | 4.57* |
| Low Mismatch vs. Low Match | .40 | .88 | .90 |
| Balanced vs. Low Match | .40 | .93 | 3.42* |
| Experiment 2 |  |  |  |
| Low Mismatch vs. Balanced | .24 | .90 | 9.34* |
| Low Mismatch vs. Low Match | .41 | .90 | 1.14 |
| Balanced vs. Low Match | .41 | .95 | 8.28* |
| Experiment 3 |  |  |  |
| Low Mismatch vs. Balanced | .24 | .86 | 10.07* |
| Low Mismatch vs. Low Match | .39 | .86 | 2.75 |
| Balanced vs. Low Match | .39 | .92 | 6.60 |

*Note:* *Indicates a significant difference.

**Supplementary Table 2.** Lower and upper ROC curve overlap boundaries used for each partial area under the curve (pAUC) analysis of subject category (non-professional, bar security, or access security) in the low mismatch prevalence condition, including *D* value for each comparison. For Experiment 1, *α* = .05, for Experiments 2 and 3, *α* = .017.

| Partial ROC Boundaries | | | |
| --- | --- | --- | --- |
| Comparisons | Lower | Upper | D |
| Experiment 1 |  |  |  |
| Professionals vs. Nonprofessionals | .17 | .87 | .22 |
| Experiment 2 |  |  |  |
| Bar Security vs. Non-Professionals | .12 | .90 | 3.29* |
| Bar Security vs. Access Security | .15 | .86 | 1.61 |
| Non-Professionals vs. Access Security | .15 | .86 | 1.65 |
| Experiment 3 |  |  |  |
| Bar Security vs. Non-Professionals | .40 | .82 | .75 |
| Bar Security vs. Access Security | .40 | .82 | 1.54 |
| Non-Professionals vs. Access Security | .14 | .87 | 1.19 |

*Note:* *Indicates a significant difference.
